# MicroRNA-146a Is a Wide-Reaching Neuroinflammatory Regulator and Potential Treatment Target in Neurological Diseases

**DOI:** 10.3389/fnmol.2020.00090

**Published:** 2020-06-05

**Authors:** Weihao Fan, Chunmei Liang, Mingqian Ou, Ting Zou, Furong Sun, Haihong Zhou, Lili Cui

**Affiliations:** ^1^Guangdong Key Laboratory of Age-Related Cardiac and Cerebral Diseases, Affiliated Hospital of Guangdong Medical University, Zhanjiang, China; ^2^Department of Neurology, Affiliated Hospital of Guangdong Medical University, Zhanjiang, China

**Keywords:** microRNA-146a, neurological diseases, single-nucleotide polymorphisms, neuroinflammation, biomarker

## Abstract

Progressive functional deterioration and loss of neurons underlies neurological diseases and constitutes an important cause of disability and death worldwide. The causes of various types of neurological diseases often share several critical nerve-related cellular mechanisms and pathological features, particularly the neuroinflammatory response in the nervous system. A rapidly growing body of evidence indicates that various microRNAs play pivotal roles in these processes in neurological diseases and might be viable therapeutic targets. Among these microRNAs, microRNA-146a (miR-146a) stands out due to the rapid increase in recent literature on its mechanistic involvement in neurological diseases. In this review, we summarize and highlight the critical role of miR-146a in neurological diseases. MiR-146a polymorphisms are associated with the risk of neurological disease. Alterations in miR-146a expression levels are crucial events in the pathogenesis of numerous neurological diseases that are spatially and temporally diverse. Additionally, the target genes of miR-146a are involved in the regulation of pathophysiological processes in neurological diseases, particularly the neuroinflammatory response. In summary, miR-146a mainly plays a critical role in neuroinflammation during the progression of neurological diseases and might be a prospective biomarker and therapeutic target. Understanding the mechanisms by which miR-146a affects the neuroinflammatory response in different neurological injuries, different cell types, and even different stages of certain neurological diseases will pave the way for its use as a therapeutic target in neurodegenerative diseases.

## Highlights

- Alterations in the microRNA-146a (miR-146a) level are common events in the pathogenesis of most neurological diseases.
- The changes in the miR-146a level in different neurological diseases or different stages of the same disease are inconsistent.
- Increasing the miR-146a level reverses the process of most neurological diseases regardless of changes in endogenous miR-146a expression.
- MiR-146a might act as an inhibitor in most neurological diseases mainly through modulation of the inflammatory response.

## Introduction

Progressive deterioration of neuronal function and loss underlies neurological diseases and constitutes an important cause of disability and death worldwide (Karikari et al., 2018). The main mechanisms of these neurological diseases, which are caused by complicated and variable processes, can be divided into several overall categories, including vascular, traumatic, epileptic, degenerative, genetic, infectious, and tumor disorders. Despite considerable efforts, the exact causes of various types of neurological diseases remain largely unknown due to the complexity of their different etiologies. However, undoubtedly, these types of diseases often share common nerve-related cellular mechanisms and pathological features, such as neuroinflammation through the activation of astrocytes and microglia as well as neurotoxicity and oxidative injury caused by dysfunctional glutamate neurotransmission; these pathological features are usually attributed to abnormal gene regulation in the nervous system (Brambilla, 2019; Gonçalves-Ribeiro et al., 2019). In particular, a rapidly growing body of evidence indicates that microRNAs (miRNAs) play pivotal roles in neurological diseases and could be amenable to therapeutic targeting (Greenberg and Soreq, 2014). Among these miRNAs, microRNA-146a (miR-146a) has gradually become prominent, based on recent literature establishing its close relationship to neurological diseases. MiR-146a plays a role in the regulation of inflammation and the innate immune system (Taganov et al., 2006; Sonkoly et al., 2008; Li et al., 2011). Moreover, it is deregulated in numerous inflammation-related diseases and pathological processes, especially in the nervous system, suggesting its important role in aspects of neurological disease, particularly neuroinflammation (Shomali et al., 2017; Juźwik et al., 2019). Furthermore, in addition to its regulatory function in inflammatory pathways, miR-146a is also involved in other physiological and pathological processes, such as apoptosis (Zhou et al., 2016), migration (He et al., 2018), growth (Boese et al., 2016), and viral infection (Sharma et al., 2015). Furthermore, numerous lines of genetic evidence suggest that polymorphisms in miR-146a can influence its function and thereby contribute to susceptibility to several neurological diseases (Cui et al., 2014, 2015). This review article summarizes the most recent findings regarding the expression levels, target genes, and mechanistic roles of miR-146a in different neurological diseases and addresses its vital role in different neurological diseases and its potential as a novel therapeutic target.

## Roles of miR-146a in Neurological Diseases

**Figure 1:**
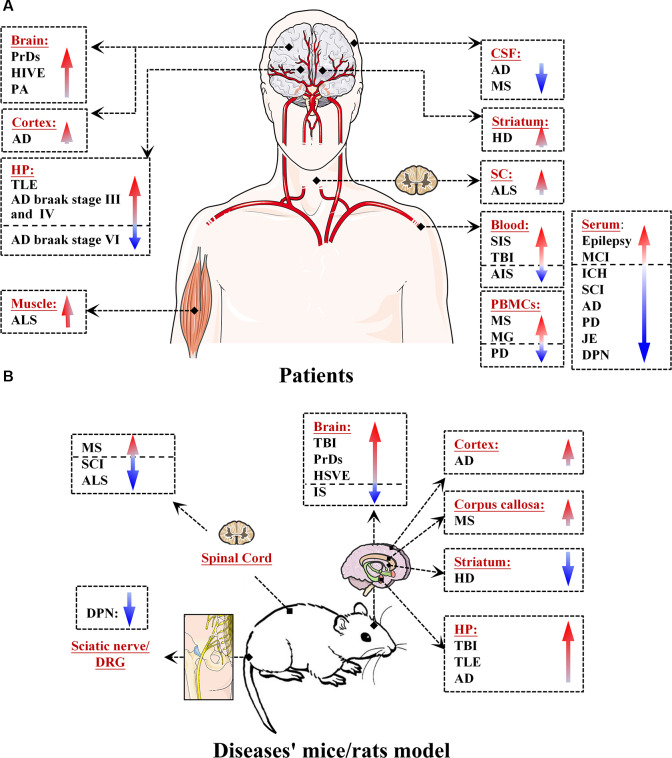
Expression levels of microRNA-146a (miR-146a) in patients and in mouse/rat models of neurological diseases. **(A)** Expression of miR-146a in the brain, cortex, hippocampus (HP), cerebrospinal fluid (CSF), spinal cord (SC), blood, serum, peripheral blood mononuclear cells (PBMCs), and muscle of patients with neurological diseases. **(B)** Expression of miR-146a in the brain, cortex, HP, striatum, corpus callosum, SC, sciatic nerve, and dorsal root ganglion (DRG) neurons of model mice/rats with neurological diseases.

**Figure 2:**
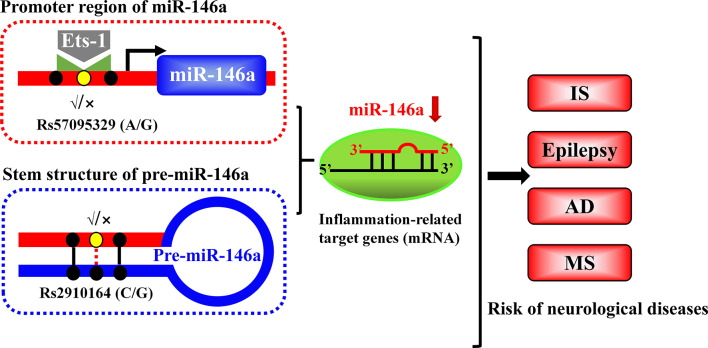
Schematic illustration of the mechanism through which two single-nucleotide polymorphisms (SNPs) alter the expression of miR-146a. The rs2910164 polymorphism results in a mismatch in the stem region of pre-miR146a and influences the expression of mature miR-146a. Another polymorphism, rs57095329, is located in the promoter region of miR-146a and affects its availability and affinity for binding to Ets-1, thereby modulating its expression.

**Figure 3:**
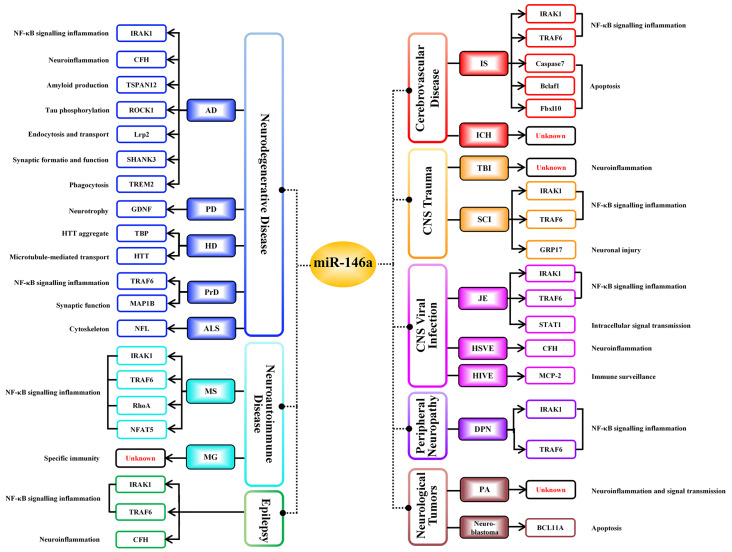
Negative regulatory targets of miR-146a in various neurological diseases. Different target genes of miR-146a have been identified in distinct neurological diseases; miR-146a negatively regulates all targeted downstream genes shown in this figure, ultimately influencing the progression of neurological diseases.

**Figure 4:**
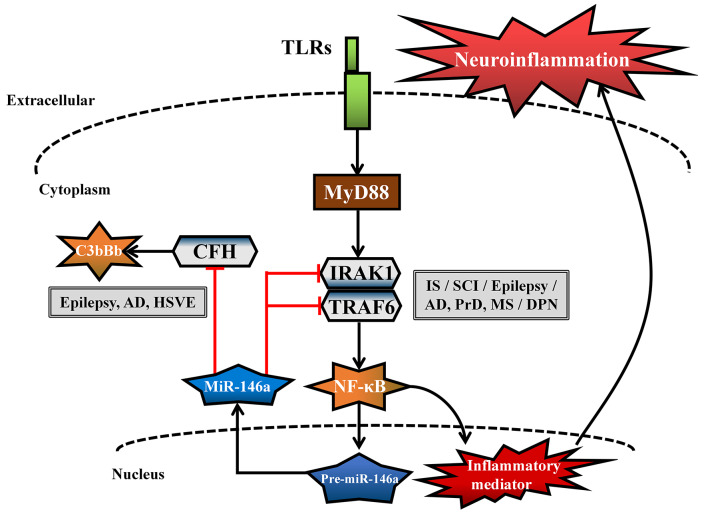
Common neuroinflammatory pathways in different neurological diseases regulated by miR-146a. The common genes [interleukin-1 receptor-associated kinase-1 (IRAK1), tumor necrosis factor receptor-associated factor 6 (TRAF6), complement factor H (CFH)] and related pathways [Toll-like receptor (TLR) signaling pathway and complement activation pathway] regulated by miR-146a are involved in the neuroinflammatory response in different neurological diseases.

MiR-146a is one of the most abundant miRNAs expressed in the central nervous system (CNS) and undoubtedly exhibits dysfunction in different types of neurological diseases and even different stages of the same neurological disease (Wang J. et al., 2015; Müller et al., 2016; Ma et al., 2019; Figure 1). Many convincing lines of evidence indicate that miR-146a is closely related to the regulation of almost all major neurological diseases, including cerebrovascular disease, CNS trauma, neurodegenerative diseases, neuroautoimmune diseases, CNS viral infections, peripheral neuropathy, and neurological tumors (Li et al., 2011; Deng et al., 2017; Liu et al., 2017; Su et al., 2017; Zhang et al., 2017; He et al., 2018; Li S. H. et al., 2018). In addition, the two most important single-nucleotide polymorphisms (SNPs) in miR-146a, rs2910164, and rs57095329, can influence the level of mature miR-146a and are associated with the risk of several important neurological diseases, such as ischemic stroke (IS), epilepsy, Alzheimer’s disease (AD), and multiple sclerosis (MS; Cui et al., 2014, 2015; Li Y. et al., 2015; Li et al., 2016; Zhang B. et al., 2015; Zhou et al., 2018; Zou et al., 2018; Labib et al., 2019). The rs2910164 (C/G) polymorphism is located in the stem region of pre-miR-146a, and the mismatch in the stem structure dysregulates the expression of mature miR-146a (Cui et al., 2014). The rs57095329 (A/G) polymorphism is located in the promoter region of miR-146a, and the risk allele might reduce the expression of mature miR-146a by altering the binding affinity of miR-146a to V-Ets oncogene homolog 1 (Ets-1), which might contribute to disease susceptibility (Cui et al., 2015; Figure 2). This evidence for the association of functional SNPs in miR-146a with neurological disorders also supports the importance of this miRNA in neurological diseases from a genetic perspective. Mechanistically, miR-146a may form a highly cooperative regulatory network with its target genes in various neuropathological processes (Figure 3), mainly regulating inflammation and innate immunity in the nervous system (Figure 4), and it is also involved in the differentiation or apoptosis of nerves (Sonkoly et al., 2008; Zhou et al., 2016). Thus, miR-146a ultimately affects the occurrence and development of neurological diseases.

### miR-146a and Cerebrovascular Disease

IS, or cerebral infarction, is the most common form of stroke and results from acute cerebrovascular events and hypoxic–ischemic encephalopathy (Dirnagl et al., 1999). MiR-146a is upregulated in the blood of patients with subacute IS (SIS; Li S. H. et al., 2015), while the opposite result has been observed in patients with acute IS (AIS; Li et al., 2017). Intracerebral hemorrhage (ICH) is one of the most severe types of stroke and has high morbidity and mortality rates (Balami and Buchan, 2012). MiR-146a levels are significantly reduced in the sera of ICH patients (Zhu et al., 2015; Hu et al., 2018) and are similar to those in the blood of AIS patients. This inconsistency may be due to different stages of the disease. Moreover, miR-146a is downregulated in endothelial progenitor cells (EPCs) of mice after middle cerebral artery occlusion (MCAO; Su et al., 2017) and in oxygen-glucose deprivation and reperfusion (OGD/R)-induced human SK-N-SH cells (Zhou et al., 2016). The above findings suggest that miR-146a may be dysfunctional in the pathological process of IS.

Overwhelming evidence indicates that neuroinflammation is an important contributor to IS (Zhang et al., 2012; Su et al., 2017). MiR-146a is abundantly expressed in neurons, microglial cells, and astrocytes, where it acts as a negative feedback regulator of inflammation through the regulation of a series of related genes; its regulation of the nuclear factor kappa-B (NF-κB) signaling pathway is the most frequently reported activity (Taganov et al., 2006; Li et al., 2011). Toll-like receptor (TLR) activation is induced at the site of vascular fibrin/fibrinogen accumulation during IS (Zhang et al., 2012), and pre-miR-146a gene expression is positively regulated by the NF-κB transcription factor. However, mature miRNA-146a acts in a negative feedback loop by inhibiting interleukin (IL)-1 receptor-associated kinase-1 (IRAK1) and tumor necrosis factor receptor-associated factor 6 (TRAF6). These two targets are upstream of the NF-κB signaling pathway, and their regulation further inhibits the expression of NF-κB target genes, such as IL-6, IL-8, IL-1β, and tumor necrosis factor alpha (TNF-α; Taganov et al., 2006; Li et al., 2011; Figure 4). MiR-146a overexpression not only inhibits cerebrovascular TLR signaling pathways to relieve neuroinflammation (Zhang et al., 2012) but also promotes the proliferation and migration of mouse EPCs to stimulate reendothelialization of damaged blood vessels and revascularization of ischemic tissues (Su et al., 2017). In addition, miR-146a contributes to the differentiation of rat oligodendrocyte progenitor cells (OPCs) into myelin oligodendrocytes (OLs) and the differentiation of neural progenitor cells into neurons by decreasing the expression of IRAK1 and TRAF6, which is beneficial for neural restoration and attenuation of ischemic brain damage in IS. However, in contrast to the positive effects of miR-146a on IS described above, the downregulation of miR-146a has been associated with an antiapoptotic role (Zhou et al., 2016; Li et al., 2017). Downregulation of miR-146a was reported to protect neurons against apoptosis in ischemic injury by targeting and promoting the expression of the antiapoptotic factor Fbxl10 (Li et al., 2017) and the proapoptotic genes Caspase7 and Bcl-2-associated transcription factor 1 (Bclaf1; Zhou et al., 2016) in an AIS cell model.

Overall, although the level of endogenous miR-146a has been reported to be inconsistent across IS subtypes, upregulation of miR-146a can reduce neuroinflammation and promote angiogenesis, thus playing a reparative role in areas of cerebral infarction in IS. However, miR-146a has also been reported to serve as a proapoptotic factor that aggravates apoptosis in AIS *in vitro*, suggesting potential side effects, but this conclusion needs further support from *in vivo* studies. Whether miR-146a can be used as a protective agent against IS and at what stage miR-146a intervention can play the optimal role need further exploration.

### miR-146a and CNS Trauma

#### Traumatic Brain Injury

Traumatic brain injury (TBI) is a common cause of death and disability worldwide. After primary injury, a group of abnormal biochemical cascades called secondary injury and involving processes, such as inflammation, oxidative stress, excitotoxicity, and apoptosis, may occur due to cell death directly caused by physical damage; these cascades result in chronic brain injury (Singh et al., 2006; Ziebell and Morganti-Kossmann, 2010). The expression level of miR-146a is significantly increased in the peripheral blood of TBI patients and is similar to that in the brains of mice in a model of TBI (Wang W. X. et al., 2015; Johnson et al., 2017; Ma et al., 2019). Moreover, elevated miR-146a levels were linked to the downregulation of the inflammatory factors IL-6 and IL-1β in the TBI mouse model (Johnson et al., 2017), suggesting a negative correlation between miR-146a and neuroinflammation in TBI. However, the specific mechanism regulating this effect remains unclear.

#### Spinal Cord Injury

Spinal cord injury (SCI) is a type of highly disabling CNS injury caused by trauma. Structural injury of the spinal cord (SC) leads to an inflammatory response, immune injury, and apoptosis of the injured SC tissue, which in turn produces SC dysfunction (Dumont et al., 2001). The level of miR-146a is lower in the sera of SCI patients than in that of controls (Paim et al., 2019). Additionally, the miR-146a level was reduced in the SC of a rat model of SCI (He et al., 2018; Tan et al., 2018) but elevated in the maintenance period (3–7 days) after SCI (Wei et al., 2016). We thus infer that miR-146a likely inhibits the inflammatory reaction *via* self-regulation at the early stage of SCI in this rat model, but after the miR-146a level decreases below a certain threshold, the body upregulates miR-146a to achieve homeostasis. An elevated level of miR-146a was reported to inhibit the IRAK1 and TRAF6 genes to decrease the secretion of proinflammatory cytokines, which promoted nerve regeneration in an SCI rat model (Tan et al., 2018). Moreover, upregulated miR-146a inhibits the expression of G-protein-coupled receptor 17 (GPR17) and thus antagonizes neuronal apoptosis in the SCI rat model (He et al., 2018). As suggested by the most recent evidence, miR-146a might function as a protective factor in SCI by suppressing inflammation and neuronal apoptosis and promoting nerve regeneration.

### miR-146a and Epilepsy

Epilepsy, which is caused by abnormal and synchronous neuronal discharges in the brain, is a chronic neurological disorder characterized by recurrent seizures (Henshall, 2014). The miR-146a level is appreciably increased in the sera of patients with epilepsy (Roncon et al., 2015; Wang J. et al., 2015), the hippocampi of patients with temporal lobe epilepsy (TLE; Aronica et al., 2010), and an experimental rat model of TLE (Roncon et al., 2015), indicating that miR-146a might be a potential biomarker for epilepsy.

Neuroinflammation and apoptosis in the brain are common but critical mechanisms involved in the pathophysiology of seizures (Kondratyev and Gale, 2000; Vezzani et al., 2011). Upregulated miRNA-146a is involved in apoptosis and the inflammatory response, reflecting the inflammation severity in status epilepticus (SE) rats (Luo et al., 2017; Zhang et al., 2018). Furthermore, intranasal delivery of miR-146a mimics relieved neuroinflammation *via* TLR signaling pathways, thus alleviating the acute phase of seizures and hippocampal damage, in a TLE rat model (Tao et al., 2017). However, in contrast to the above view, complement factor H (CFH), which potently inhibits the amplification of the alternative pathway of complement activation (Makou et al., 2013), can be downregulated by upregulation of miR-146a expression (Figure 4), thus contributing to IL-1β upregulation in a TLE rat model (Li T. R. et al., 2018).

In brief, evidence consistently shows that the level of miR-146a is upregulated during TLE. Although reports are inconsistent, targeting miR-146a for intervention seems to be able to influence the occurrence of epilepsy. However, the optimal doses and time points for intervention need to be identified, and systematic behavioral and pathological evaluations of seizures *in vivo* need to be conducted.

### miR-146a and Neurodegenerative Diseases

#### Alzheimer’s Disease

AD is an age-related progressive neurodegenerative disease that causes dementia symptoms, including memory loss and cognitive impairment (McKhann et al., 1984). The miR-146a level is increased in the sera of patients with mild cognitive impairment (MCI) but decreased in patients with AD (Dong et al., 2015; Swarbrick et al., 2019), indicating that circulating miR-146a might be a potential biomarker for AD. In addition, in patients with AD, miR-146a is upregulated in the neocortex and hippocampus (Sethi and Lukiw, 2009; Clement et al., 2016; Jaber et al., 2017; Swarbrick et al., 2019) but downregulated in the CSF (Müller et al., 2014, 2016). Interestingly, the relative expression of miR-146a has been reported to vary in patients with different Braak stages of AD. For instance, the miR-146a level is increased in the hippocampi of patients with Braak stage III and IV AD but decreased in patients with Braak stage VI AD (Müller et al., 2014), suggesting that the miR-146a level is dynamic during the progression of AD.

The role of miR-146a in AD-related neuroinflammation remains controversial. This role is mainly manifested in the antagonization of TLR signaling pathways by overexpressed miR-146a, which alleviates pathological processes, including neuroinflammation, glial activation, Aβ deposition, and tau phosphorylation, in the hippocampus in a mouse model of AD and thus rescues cognitive impairment (Li et al., 2011; Mai et al., 2019). However, although the above experiments support the protective effect of miR-146a overexpression in AD, several lines of evidence suggest that miR-146a not only fails to alleviate inflammation but also inhibits CFH, thus enhancing proinflammatory effects on neurons *in vitro* (Lukiw et al., 2008; Pogue et al., 2009). The above contradictory experimental results may be due to the limitations of different experimental models; thus, more experiments *in vivo* to prove the role of miR-146a and these inflammation-related target genes in AD are needed. In addition to playing an inflammatory regulatory role, miR-146a exerts different effects by targeting noninflammation-related target genes during AD. MiR-146a is reported to negatively regulate tetraspanin 12 (TSPAN12; Li et al., 2011; Lukiw, 2012), and insufficient TSPAN12 increases neurotoxic Aβ42 deposition by inhibiting the hydrolysis of amyloid precursor protein (APP) in primary human neuronal cell–glial cell cocultures (Li et al., 2011; Seipold and Saftig, 2016). Moreover, miR-146a negatively regulates rho-associated coiled-coil containing protein kinase 1 (ROCK1) protein translation in neural SH-SY5Y cells, which results in decreased phosphorylation of phosphatase and tensin homolog (PTEN) and thus promotion of tau hyperphosphorylation and cytoskeleton destruction (Wang et al., 2016). Low-density lipoprotein receptor-related protein-2 (Lrp2) has vital functions in endocytosis and transport and plays protective roles in AD (Marzolo and Farfán, 2011). MiR-146a has been reported to target Lrp2 and reduce its expression, leading to decreased activation of protein kinase B (Akt) and induction of proapoptotic caspase-3 expression, which results in increased neuronal apoptosis *in vitro* (Zhang et al., 2016). SHANK3 is a multidomain synaptic and scaffold protein whose deficiency leads to synapse loss (Jaber et al., 2017), and a negative relationship between miR-146a and SHANK3 has been observed in the brain tissues of patients with AD (Pham et al., 2010; Jaber et al., 2017). Furthermore, miR-146a expression is inversely correlated with triggering of receptor expression on myeloid cells 2 (TREM2) expression in patients with AD (Zhao et al., 2016), which might eliminate the ability of microglial cells to effectively engulf pathological proteins through TREM2 (Yuan et al., 2016). However, although these studies have clarified the relationship between miR-146a and the abovementioned target genes *in vitro*, the specific mechanisms have not been thoroughly explored in patients with AD or animal models.

In conclusion, miR-146a forms a highly cooperative miR-146a–messenger RNA (mRNA) signaling network with its target genes that may affect amyloid production, tau phosphorylation, apoptosis, synapse formation, phagocytosis, neuroinflammation, and other pathological processes. Further *in vivo* investigations are needed to determine whether miR-146a protects patients with AD from pathological damage.

#### Parkinson’s Disease

Parkinson’s disease (PD) is a neurodegenerative disorder characterized by the death of nigrostriatal dopaminergic neurons, which results in decreased levels of striatal dopamine, resting tremors, muscle stiffness, bradykinesia, unstable posture, and other symptoms (Sveinbjornsdottir, 2016). The miR-146a level is decreased in peripheral blood mononuclear cells (PBMCs) and sera of patients with PD (Ma et al., 2016; Caggiu et al., 2018). However, few studies have explored the function of miR-146a in PD. Glial-cell-derived neurotrophic factor (GDNF) is a glia-related protein that promotes the survival and function of nigrostriatal dopamine neurons and their neurite growth while protecting against neurodegeneration through the inhibition of neuroinflammation (Lin et al., 1993; Rocha et al., 2012). MiR-146a has been reported to be able to target and regulate GDNF, which may in turn influence the activation of glia and ultimately contribute to PD progression (Kumar et al., 2015). However, further evidence regarding the mechanism of miR-146a in PD is needed.

#### Huntington’s Disease

Huntington’s disease (HD) is an autosomal dominant progressive neurodegenerative disease involving the loss of neurons in the cortex and striatum caused by amplification of the CAG trinucleotide repeat length in the Huntingtin (HTT) gene (Imarisio et al., 2008). MiR-146a is upregulated in the striatum of patients with HD (Martí et al., 2010), but decreased levels of miR-146a have been reported in the striatum in a mouse model of HD and an HD cell model (Sinha et al., 2010, 2011; Ghose et al., 2011). The increased expression of TLR4 in the striatum of patients with HD is related to the clinical progression and pathological state of HD (Vuono et al., 2020). However, although miR-146a has been reported to exert a protective effect in HD, no clear evidence of neuroinflammation related to the effect of miR-146a on HD has been found. The interaction of mutant HTT aggregates with several transcription factors, such as p53, NF-κB, and TATA-binding protein (TBP), and the recruitment of these transcription factors might cause transcriptional dysregulation (Cha, 2007; Sinha et al., 2010; Ghose et al., 2011). Elevated p53 increases mutant HTT aggregation and inhibits NF-κB activity and miR-146a expression in mouse and cell models of HD (Ghose et al., 2011), and a reduction in the miR-146a level upregulates TBP in the STHdh^Q111^ cell lines (Sinha et al., 2010), all of which might contribute to HD. Heat shock proteins (HSPs) are antagonist proteins that protect against HTT-mediated toxicity, which is regulated by heat shock factor 1 (HSF1; Das and Bhattacharyya, 2015). HSF1 can promote the expression of miR-146a, which targets HTT and thereby inhibits the aggregation of mutant HTT *in vitro* (Sinha et al., 2011). Based on the current limited evidence, miR-146a appears to exert a protective effect in HD progression.

#### Prion Diseases

Prion diseases (PrDs) are a group of fatal neurodegenerative diseases that impact the nervous systems of humans and animals. The evolution of PrDs is attributed to the misfolding of normal cellular prion protein (PrP^C^) into a disease-associated conformation (PrP^Sc^) that is uniquely transmissible between individuals (Whitechurch et al., 2017). MiR-146a levels are elevated in the brains of patients with different PrDs, such as Creutzfeldt–Jakob disease (CJD) and Gerstmann–Straussler–Scheinker (GSS) syndrome, as well as in mouse models of scrapie (Lukiw et al., 2011; Saba et al., 2012; Boese et al., 2016).

Pathological neuroinflammation is the core of PrD progression (Carroll and Chesebro, 2019), which is characterized by PrP^Sc^ aggregates, gliosis hyperplasia, and increased neuronal death, which stimulates spongiform changes (Hughes and Halliday, 2017). Upregulated miR-146a impairs the activation of microglial cell proinflammatory phenotypes by inhibiting TLR signaling pathways and might result in the limitation of excessive neuroinflammation in rodent models (Saba et al., 2012). Moreover, PrDs also cause synapse loss and dysfunction. Dendritic microtubule-associated protein 1B (MAP1B) is required for dendritic spine development, and the increased miR-146a expressed in the brains of scrapie model mice might target dendritic MAP1B protein and induce synaptic dysfunction (Chen and Shen, 2013; Boese et al., 2016). In brief, modulation of miR-146a may have a dual effect in PrD, increasing the host immune response to target misfolded protein aggregates and increasing dendritic MAP1B protein levels, thereby altering synaptic transmission.

#### Amyotrophic Lateral Sclerosis

Amyotrophic lateral sclerosis (ALS) is a neurodegenerative disease caused by selective degeneration of the upper and lower motor neurons (UMNs and LMNs, respectively) in the brain and SC (Gordon, 2013). Cu/Zn superoxide dismutase 1 (SOD1) mutations and protein misfolding are associated with inflammatory and neurotoxic pathways in ALS (McCombe and Henderson, 2011). MiR-146a is elevated in the SC and muscle in patients with ALS (Campos-Melo et al., 2013; Pegoraro et al., 2017), and the elevated expression of miR-146a likely inhibits the reduction in muscle mass and the production of inflammatory cytokines by antagonizing NF-κB activation (Pegoraro et al., 2017). In addition, miR-146a is downregulated in the SCs of mice expressing mutant SOD1 (mSOD1; Cunha et al., 2018). Neurotoxic astrocytes are reportedly increased in the cortexes of mSOD1-expressing mice (Gomes et al., 2019), and mSOD1 can promote the transformation of microglial cells into cells with a proinflammatory M1 phenotype (Cunha et al., 2018). In the pathology of ALS, miR-146a deficiency might be associated with the transformation of astrocytes and microglial cells into neurotoxic and proinflammatory phenotypes, which might contribute to MN degeneration (Gomes et al., 2019). These results indicate that miR-146a overexpression exerts a protective effect in ALS. However, neurofilaments (NFLs), proteins that maintain neuronal morphology, are decreased in response to miR-146a upregulation in the SCs of patients with ALS (Campos-Melo et al., 2013), suggesting a side effect of targeting miR-146a. In summary, miR-146a plays a regulatory role in the progression of ALS. This role involves inhibition of neuroinflammation, maintenance of skeletal muscle homeostasis, and effects on glial cell transformation.

### miR-146a and Neuroautoimmune Diseases

#### Multiple Sclerosis

MS is a common demyelinating CNS disease characterized by alterations in innate immunity, chronic CNS inflammation, progressive demyelination, and axonal loss (Devier et al., 2015). MiR-146a expression in CSF is significantly reduced in MS patients compared with controls (Quintana et al., 2017) but elevated in PBMCs of patients with relapsing–remitting multiple sclerosis (RRMS; Waschbisch et al., 2011) and in the corpus callosa (Zhang et al., 2017) and SCs of mice with experimental autoimmune encephalomyelitis (EAE; Wu et al., 2015).

The blood–brain barrier (BBB) is impaired by the migration of substantial numbers of leukocytes to the CNS in MS (Larochelle et al., 2011), and miR-146a can suppress NF-κB activity in brain endothelial cells (BECs) by inhibiting nuclear factor of activated T cells 5 (NFAT5), RhoA, IRAK1, and TRAF6 and subsequently negatively regulate the adhesion of leukocytes to BECs *in vivo* and *in* *vitro* (Wu et al., 2015). In addition, overexpressed miR-146a promotes the differentiation of OPCs into OLs and enhances remyelination by inhibiting TLR signaling pathways in mice with EAE (Santra et al., 2014). Furthermore, microglial cells with the M2 phenotype can phagocytose and remove damaged myelin sheaths and secrete growth factors to promote remyelination (Gudi et al., 2014). Overexpression of miR-146a increases the polarization of microglial cells toward the M2 phenotype in mice with EAE, which ultimately exerts beneficial effects in MS (Zhang et al., 2017). In summary, the current literature indicates that miR-146a might play a protective role in MS by inhibiting neuroinflammation and promoting remyelination.

#### Myasthenia Gravis

Myasthenia gravis (MG), a neuroautoimmune disease characterized by abnormal activation of B and T lymphocytes in the immune system, is caused by autoantibodies against synaptic membrane antigens (mainly nAChR) in neuromuscular junctions (Miranda et al., 2017). MiR-146a is significantly upregulated in PBMCs of patients with MG (Lu et al., 2013) and in activated B cells in response to AChR antigen (Zhang J. et al., 2015). Inhibition of miR-146a decreases the numbers of AchR-specific B cells, which secrete anti-AchR antibodies in mice with experimental autoimmune myasthenia gravis (EAMG; Lu et al., 2013; Vrolix et al., 2014; Zhang J. et al., 2015). Furthermore, dendritic cells (DCs) are one of the most effective antigen-presenting cell types in the immune system and continuously produce exosomes during maturation (Segura et al., 2005). Overexpression of miR-146a in DC exosomes can reduce T lymphocyte proliferation and polarize T helper cells toward an anti-inflammatory phenotype, thereby alleviating the clinical symptoms of MG in mice with EAMG (Yin et al., 2017). The abovementioned studies indicate that regulating the expression of miR-146a in B and T lymphocytes is expected to alleviate the progression of MG. However, more evidence is needed to explore the specific target genes of miR-146a in MG.

### miR-146a and CNS Viral Infections

Japanese encephalitis virus (JEV) is a neurotropic mosquito-borne flavivirus that causes Japanese encephalitis (JE) in humans (Thongtan et al., 2010). The level of miR-146a is reduced in the sera of patients with JE (Baluni et al., 2018). Activation of microglial cells by JEV results in inflammation-induced neuronal damage, whereas JEV simultaneously persists in microglial cells, which makes these cells a potentially threatening virus repository (Thongtan et al., 2010). MiR-146a expression is increased in microglial cells of BALB/c mice after infection with JEV (Sharma et al., 2015; Deng et al., 2017). The increased miR-146a inhibits the activation of TLR signaling pathways, thus reducing the secretion of proinflammatory cytokines. In addition, JEV-induced upregulation of miR-146a also leads to suppression of its target STAT1, which results in abolition of Jak-STAT pathway activity and reduced expression of interferon-stimulated genes (ISGs) in human brain microglial cells (i.e., CHME3 cells). The above results indicate that, although miR-146a suppresses host inflammatory responses, it simultaneously creates a cellular environment favorable for the survival of JEV (Sharma et al., 2015; Deng et al., 2017).

Herpes simplex virus (HSV) usually establishes a lytic infection in epithelial cells, resulting in blisters or ulcers; subsequently, the virus becomes latent, resides within the trigeminal ganglia, and induces herpes simplex encephalitis (HSVE), which can also cause neuroinflammation, leading to neuronal death and dysfunction (Whitley, 2006). Mice infected with HSV show increased miR-146a levels in their brains (Majer et al., 2017). The elevation of miR-146a expression in HSV-infected human neural cells might contribute to viral evasion of complement by targeting CFH and to the promotion of neuropathological changes by activating the arachidonic acid cascade (Hill et al., 2009).

Human immunodeficiency virus (HIV) infection is usually associated with chronic inflammation from human immunodeficiency encephalitis (HIVE) in the brain, and macrophages and microglia are the main reservoirs of HIV (Del Valle and Piña-Oviedo, 2006). MiR-146a expression is increased in the brains of patients with HIVE, particularly in primary human fetal microglial cells infected with HIV-1 (Rom et al., 2010). The proinflammatory chemokine MCP-2, which is secreted by microglial cells, plays an inhibitory role in HIV entry and replication (Yang et al., 2002), and miR-146a overexpression prevents HIV-induced secretion of MCP-2 and regulates the spread of HIV in the brain, indicating that miR-146a inhibits inflammation but sustains the survival of HIV, thus maintaining HIV-mediated chronic encephalitis (Rom et al., 2010). The current evidence generally demonstrates that the elevation of miR-146a expression caused by virus infection might decrease neuroinflammation in the CNS, does not impede viral replication, and promotes immune escape of the virus.

### miR-146a and Peripheral Neuropathy

Diabetic peripheral neuropathy (DPN) is a severe chronic complication of type 2 diabetes mellitus (T2DM) that manifests as chronic distal symmetry in sensory and motor multiple neuropathies (Pop-Busui et al., 2017). The miR-146a level is significantly reduced in the sera of T2DM patients (Baldeón et al., 2014) and is also downregulated in sciatic nerve tissues and dorsal root ganglion (DRG) neurons in mouse and rat models of diabetes (Wang et al., 2014; Jia et al., 2016; Liu et al., 2017). The inflammatory response is involved in the pathogenesis of DPN (Pop-Busui et al., 2017). As unique glial cells, Schwann cells can secrete and express a variety of neurotrophic factors in the peripheral nervous system and thus play a role in preventing neuronal cell death and accelerating axonal myelination (Vincent et al., 2011). MiR-146a overexpression decreases the inflammatory response and apoptosis of Schwann cells in hyperglycemic rats, which might contribute to reversing the pathological process of DPN (Luo et al., 2019). In addition, miR-146a mimics reportedly attenuate hyperglycemia-mediated inflammation and the apoptosis of DRG neurons (Wang et al., 2014), promoting axonal growth in neurons by reducing the expression of IRAK1, TRAF6, and caspase-3 *in vitro* and thereby alleviating diabetic sensory neuropathy (Jia et al., 2016). In summary, the current evidence indicates that miR-146a does not significantly reduce the level of glucose in the blood of DPN patients but instead affects the progression of DPN mainly by inhibiting neuroinflammation and apoptosis. This observation suggests a role for this miRNA as a therapeutic target in DPN.

### miR-146a and Neurological Tumors

Pilocytic astrocytoma (PA) is the most frequent CNS tumor in young patients (Collins et al., 2015). MiR-146a is significantly upregulated in the brains of children with PA and negatively regulates senescence-related inflammatory responses and the cell cycle *via* the NF-κB and extracellular regulated protein kinase/mitogen-activated protein kinase (ERK/MAPK) pathways (Jones et al., 2015). Neuroblastoma (NB), one of the most common extracranial tumors of neuroectodermal cells in children, originates from the developing sympathetic nervous system (Cheung and Dyer, 2013). Apoptosis is a suppressive element in the growth and survival of human NB, and BCL11A encodes a C2H2 zinc finger transcription factor that can reduce apoptosis (Nakamura et al., 2000). MiR-146a might function as an NB suppressor by targeting BCL11A to inhibit the growth of human NB cells and induce their apoptosis *in vitro* (Li S. H. et al., 2018). In summary, miR-146a might affect the progression of neurological tumors by regulating inflammation and transcription, but the role of miR-146a in the abovementioned neurological tumors is not well understood.

## Discussion and Perspective

In this review, we highlight the role of miR-146a in neurological diseases and its mechanism in the regulation of pathophysiological processes, particularly regarding the neuroinflammatory response in neurological diseases (Supplementary Table S1). Our review addresses the important role of miR-146a as a target in neurological diseases.

Alterations in miR-146a levels are crucial events in the pathogenesis of numerous neurological diseases, reflecting the extensive role of miR-146a in the pathology of these diseases. Most studies of miR-146a and neurological diseases have focused on the relationship between miR-146a and neuroinflammation, suggesting the correlation between the level of miR-146a and the severity of neuroinflammation. However, notably, these changes are inconsistent at different stages of several diseases. Interestingly, miR-146a is always expressed at low levels in the acute phase of these diseases, such as AIS (Li et al., 2017) and SCI (Paim et al., 2019), and is upregulated in the chronic phase of some diseases, such as SIS (Li S. H. et al., 2015), the maintenance period of SCI (Wei et al., 2016), and Braak stages III and IV of AD (Müller et al., 2014). Currently, the observation of this phenomenon is based on inference from limited evidence, and the mechanism is still unclear. We speculate that endogenous miR-146a may be a potential inflammatory marker. During an inflammatory response, the endogenous miR-146a level often decreases or does not change significantly in response to intense neuroinflammation; however, it slowly increases during a mild chronic neuroinflammatory response. After the peak period of acute inflammation, endogenous miR-146a might play a role in the inflammatory response as an inflammatory suppressor to repair nerve damage caused by excessive inflammation. However, this hypothesis needs to be supported by additional systematic evidence.

In addition, miR-146a expression varies among tissues and fluids, such as the blood, CSF, SC, and brain, in the same neurological disease. This variation is unsurprising, given that the BBB allows the biochemical conditions in the brain to differ considerably from those in the blood. Changes in tissue-specific miR-146a levels may result from damage to the BBB (Liu et al., 2016), feedback from cells in different tissues in response to disease states, and other events. The miR-146a level in peripheral blood is more likely to be a potential marker for diseases, while the change in the miR-146a level in the brain is more likely to be a process useful for therapeutic targeting. Moreover, the expression of miR-146a may differ between human patients and model rodents with the same neurological disease due to species differences or yet-imperfect animal modeling methods; thus, the expression levels of miR-146a in human patients are much more reflective of the true clinical situation. Considering the extensive and sensitive changes in the miR-146a level in neurological diseases, further clinical data are needed to determine whether miR-146a is a biomarker for certain neurological diseases, as well as to quantify its specificity and alterations in different stages of specific diseases.

Although the above results establish that changes in the miR-146a level do not follow the same trend for all these diseases, the intervention results clearly show that miR-146a is an unquestionably potent inhibitor of neuroinflammation, strongly suggesting that it is a potential therapeutic target in many neurological diseases. Mechanistic studies have revealed that miR-146a forms a cooperative regulatory network with its target genes and that this network mainly negatively regulates the neuroinflammatory response, subsequently promoting the transformation of glial cells into anti-inflammatory phenotypes, regulating angiogenesis and neural regeneration, producing myelin protein, and exerting antiapoptotic effects (Figure 3). In addition, miR-146a participates in the neuroinflammatory response of different neurological diseases by regulating the common inflammation-related target genes (IRAK1, TRAF6, and CFH) and related pathways (TLR signaling pathway and complement activation pathway; Figure 4). However, when miR-146a binds and regulates inflammation-related target genes, it also binds noninflammation-related target genes, causing certain side effects, such as apoptosis, inducing synaptic dysfunction. Although most studies have suggested that miR-146a may improve the outcomes of neurological diseases, these potential side effects of miR-146a cannot be ignored. First, the mechanistic involvement of miR-146a—not only its intervention with neuroinflammatory pathways but also its interaction with its other target genes—differs across neurological diseases. Thus, the mechanisms and effects of miR-146a in different neurological diseases should be clarified. Second, although miR-146a intervention *in vivo* has shown beneficial effects against numerous neurological diseases in mouse models, dose evaluation, evaluation of other tissue functions, and information on the effects of long-term treatment are lacking. Third, because of the presence of the BBB, effective expression of miR-146a in the affected regions of patients with neurological diseases is another problem requiring resolution. Our group recently provided data on the effects of intranasal administration of exogenous miR-146a on cognitive impairment and seizure onset in mouse models, highlighting the application potential of the nose-to-brain route for miRNA delivery in brain-related neurological diseases (Tao et al., 2017; Mai et al., 2019).

In summary, miR-146a plays a critical role in neuroinflammation during the progression of several neurological diseases, in which miR-146a might serve as a biomarker and therapeutic target. However, considerable ambiguity remains and needs to be clarified. Understanding the functional roles of miR-146a in the regulation of neuroinflammation and in other regulatory pathways in different neurological injuries, different cell types, and different stages of certain neurological diseases will pave the way for the use of this miRNA as a therapeutic target in neurodegenerative diseases.

## Author Contributions

LC and HZ formulated the original idea and reviewed and approved the manuscript. WF drafted the manuscript and designed the table and figures. LC and CL critically revised the manuscript. MO designed the figures. TZ, MO, and FS reviewed the manuscript. All authors read and approved the final manuscript.

## Conflict of Interest

The authors declare that the research was conducted in the absence of any commercial or financial relationships that could be construed as a potential conflict of interest.
