# Supplementary material for: MicroRNA-146a Is a Wide-Reaching Neuroinflammatory Regulator and Potential Treatment Target in Neurological Diseases
Source: Front Mol Neurosci. 2020 Jun 5;13:90. doi: 10.3389/fnmol.2020.00090 (PMC7291868; doi:10.3389/fnmol.2020.00090)
Supplement: Supplementary file 1 [file Table_1.DOC]

**Table S1. Summary of miR-146a expression and the target genes of this miRNA in neurological diseases.**
